# Myoblast expansion defects leads to muscle growth delay and subsequent compensatory adaptation in adult Atrx cKO skeletal muscle

**DOI:** 10.1186/1756-8935-6-S1-P29

**Published:** 2013-03-18

**Authors:** Michael S Huh, Keqin Yan, Tina Price O’Dea, David J Picketts

**Affiliations:** 1 Regenerative Medicine Program, Ottawa Hospital Research Institute, Ottawa, Ontario, Canada, K1H8L6

## Background

The growth of muscle tissue and its regeneration from injury is crucially dependent on a self-renewing population of muscle progenitor cells called satellite cells. Activated satellite cells give rise to a rapidly expanding population of myoblasts that increase muscle mass by differentiating into new or adding into pre-existing muscle fibers. Patients with mutations in the chromatin remodeling gene *ATRX* are clinically characterised by severe cognitive disabilities and muscular hypotonia, thus dramatically compromising their independent locomotory ability.

## Materials and methods

We generated a skeletal muscle specific knockout mouse model by interbreeding *Myf5-Cre* mice with mice harbouring the *Atrx floxed* conditional knockout allele (herein referred to as Atrx cKO). Body mass of Atrx cKO and control littermates were measured along with general morphometric analysis of select hindlimb muscles at 3-weeks, 5-weeks, and greater than 8-weeks of age. Methods of analysis included single muscle fiber preparations from the extensor digitorum longus (EDL) and soleus muscles as well as RNA expression analysis from the EDL and soleus muscles. Primary myoblasts were also cultured from dissociated muscle tissue from the hindlimbs and analyzed via immuno-fluorescent microscopy. Neuromuscular acuity and endurance was assessed in Atrx cKO and control littermates by the roto-rod apparatus. Muscle strength was assessed by utilizing a digital grip strength measuring apparatus.

## Results

Atrx cKO mice presented with telltale characteristics of weaker musculature, exemplified by spinal kyphosis and reduced body mass at 3-weeks of age. Satellite cell derived myoblasts from Atrx cKO mice were incapable of rapid expansion in culture but were fully capable of terminally differentiating. Atrx cKO myoblasts displayed delayed cell cycle progression through mid-late S-phase and rampant signs of genomic instability characterised by fragmented nuclei, γ-H2AX foci, and telomeric aberrations. Despite inefficient myoblast proliferative capacity, Atrx cKO animals were able to re-establish normal body mass by adulthood. Muscular fitness and function in Atrx cKO mice was also age dependent, as younger 3-week old Atrx cKO mice had a reduced capacity to stay on the roto-rod and poorer gripping strength. However, differences in grip strength of adult Atrx cKO mice were almost indistinguishable from their control littermates. Data regarding pathways mediating the hypertrophic compensatory adaptation in Atrx cKO mice will also be presented.

## Conclusions

Inefficient expansion of activated satellite cells in Atrx cKO mice results in delayed muscle development up to 3-weeks of age, when myonuclear accretion reaches its plateau. Reduced muscle mass at 3-weeks of age also correlated with poorer performance in physical tasks that require muscular force, endurance, and coordination. Compensatory mechanisms are triggered after 3-weeks of age in Atrx cKO mice that allow for the eventual recovery of body mass and muscle functionality in adults.

